# Breast Cancer Survivors' Perceptions of Prevention versus Control of Future Cancer Recurrence

**DOI:** 10.1155/2019/2652180

**Published:** 2019-05-02

**Authors:** Monira Alwhaibi, Christa L. Lilly, Hannah Hazard, Kimberly M. Kelly

**Affiliations:** ^1^College of Pharmacy, King Saud University, Riyadh, Saudi Arabia; ^2^Medication Safety Research Chair, King Saud University, Riyadh, Saudi Arabia; ^3^School of Public Health, West Virginia University, Morgantown, WV, USA; ^4^School of Medicine, West Virginia University, Morgantown, WV, USA; ^5^West Virginia University Cancer Institute, West Virginia University, Morgantown, WV, USA; ^6^School of Pharmacy, West Virginia University, Morgantown, WV, USA

## Abstract

**Background:**

The Institute of Medicine has established Survivorship Care Planning as a critical component of cancer care to ensure that cancer survivors receive the appropriate follow-up care in a timely manner and support cancer survivors in dealing with the risk of recurrence, yet little is known about how cancer survivors think about preventing or controlling future cancer recurrence. This study sought to assess breast cancer women's perceived prevention and perceived control of future cancer recurrence.

**Methods:**

Women with a history of breast cancer (n=114) were surveyed, and data were analyzed using concurrent mixed methods. Binary logistic regression models examined predictors of perceived prevention and perceived control of cancer recurrence.

**Results:**

Most women perceived that they could control cancer recurrence (89%); few (30%) perceived that they could prevent cancer recurrence. Women reported components of the timeline (e.g., early diagnosis), identity (e.g., cancer in body), causes (e.g., hereditary), consequences (e.g., witness success), and cure/control (e.g., exercise) or lack of cure/control. Women who reported lack of control were less likely to perceive that they could control cancer recurrence. Women who reported causes were less likely to perceive that they could prevent or control cancer recurrence.

**Conclusions:**

Women's perceptions about the prevention and control of cancer recurrence are important and different factors in the minds of women with breast cancer. Most women believed they could control cancer recurrence; however, few believed they could prevent cancer recurrence. Interventions to focus on control of cancer recurrence, focusing on evidence-based clinical and lifestyle interventions, are needed.

## 1. Introduction

Breast cancer is among the top causes of disability of older women globally [1]. It accounts for 30% of the total cancer cases and 15% of cancer deaths [2, 3]. The use of more effective and less toxic medications has resulted in an increasing rate of survivorship among women with breast cancer, with the relative survival rate at 10 years after diagnosis for combined stages of breast cancer being over 80% in the United States [4]. Moreover, breast cancer survivors have a higher risk of developing a future cancer than individuals who have no prior cancer history [5, 6]. This large and growing population calls for increasing attention to clinical decision-making in the cancer care context in terms of beliefs about prevention and control of recurrence for those who survive their initial breast cancer.

The Institute of Medicine has established Survivorship Care Plans (SCPs) as a critical component of cancer care that should be provided to cancer survivors upon completion of treatment. SCPs can include follow-up screening, signs of recurrence, monitoring and managing psychosocial effects, guidelines for lifestyle modifications and health promotion activities, and empowering cancer survivors to support for their own healthcare needs [7, 8]. This information can ensure that cancer survivors receive the appropriate follow-up care in a timely manner and can support cancer survivors in dealing with the risk of recurrence [7, 8]. Further, the American Society of Clinical Oncology has established guidelines for what a Survivorship Care Plan should include for breast cancer patients [8]. Both of these reports suggest that preventing and controlling cancer recurrence is critical to optimizing outcomes in cancer survivors. The National Cancer Institute (NCI) defines cancer control as reducing the incidence, morbidity, mortality of cancer, and enhancing the quality of life of cancer survivors. Cancer control includes cancer prevention, early detection, diagnosis, and treatment [9]. Cancer prevention is an action taken to lower the chance of getting cancer, whether to prevent cancer from the initial onset or prevention of recurrence [10]. Although organizations advocate for greater information about cancer control and prevention, the lay individual's perception of prevention and control of future cancer recurrence is understudied.

The risk of cancer recurrence in breast cancer survivors has been linked to modifiable behavioral factors and unmodifiable clinical and socioeconomic factors. Behavioral factors that increase the risk of breast cancer recurrence include obesity [11], alcohol consumption [12, 13], hormone replacement therapy [14], stress [15], and low physical activity [16, 17]. Changing these behaviors can reduce the risk of recurrence [18, 19], thereby reducing breast cancer morbidity and mortality [20, 21]. Clinical factors such as tumor characteristics, hormone receptor status, and primary tumor therapy also predict cancer recurrence and do so more strongly than behavioral factors [22, 23]. Risk of cancer recurrence may be reduced for women with these clinical markers by prophylactic mastectomy and hormone therapy (e.g., tamoxifen and raloxifene) [24–26].

In the mind of breast cancer survivors', recurrence may be distilled into two distinct concerns: (1) “Will I get cancer again?” and (2) “Will I survive cancer if I get it again?” Studies have shown that cancer survivors believe that they can prevent cancer recurrence through behavioral changes [27, 28]. Some breast cancer survivors have attributed prevention of breast cancer recurrence to a positive attitude, diet, healthy lifestyle, exercise, prayer, complementary medicine, tamoxifen, luck, stress reduction, and medical screening [29, 30].

Previous research has given us a scant understanding of the factors that affect perceived prevention of cancer recurrence and perceived control of future cancer recurrence, with the exception of studies providing a list of factors that may play a role in cancer recurrence [31–34]. The current study contributes to the literature by suggesting that perception of prevention and control of cancer recurrence can be affected by several factors, including demographics, risk communication, and emotions (e.g., cancer worry), and these may be unique to prevention or control. Also perception of prevention and control of breast cancer recurrence can be affected by the patient's own representation of the illness, which may be comprised of five common attributes, including (1) identity/label (e.g., symptoms), (2) timeline of developing cancer (e.g., acute, chronic, age of onset) (3) consequences (e.g., physically, socially), (4) causes (e.g., hereditary, environmental), and (5) cure/control (i.e., which strategies can cure the illness or prevent recurrence) [35].

In addition, the current literature lacks clarity as to potential differences in women's perceptions about prevention and control of cancer recurrence. Distinguishing between cancer prevention and cancer control may be helpful in crafting messages to cancer survivors by policy makers and health authorities. For example, if cancer survivors do not believe that they can prevent cancer, messages may need to be framed in terms of cancer control.

Using a mixed methods approach, we will contribute to the quantitative and qualitative understanding of prevention and control of cancer recurrence to better compare and contrast these concepts and to aid in understanding how best to motivate health action, particularly in the clinical context. Thus, the aims of this paper are multifold: (1) to understand if women differ in perceptions about prevention and control of cancer recurrence, (2) to describe women's beliefs about prevention and control of cancer recurrence, and (3) to examine the factors that may play a role in perceptions of prevention and control of cancer recurrence.

## 2. Methods

### 2.1. Participants

Women who participated in this study met the following inclusion criteria: being 18 years of age or older, having previous history of breast cancer, having ability to read and write in English, and being seen at a university-based breast cancer clinic. Participants either were diagnosed with breast cancer within one year and currently under active treatment or were between two to five years after diagnosis. Of women who consented to participate in the study and provided medical records, 80.85% (n=114) completed and returned the survey.

### 2.2. Procedures

An informal ethnographic study was conducted with patients and clinical staff prior to initiating data collection. The corresponding author spent time observing a university-based breast oncology clinic in a Midwestern city and have conducted interviews with patients and clinical staff (e.g., oncologists, nurses, and schedulers). Based on information gathered, a survey was established, utilizing previously existing validated scales and newly constructed open-ended items to increase our understanding of perceptions among women with breast cancer [36].

The current study was approved by the institutional ethical review board from a university-affiliated breast oncology clinic in a Midwestern state. Women were approached at a university-based breast cancer clinic. Eligibility of the participant was reviewed and confirmed. Each eligible participant was asked to complete an informed consent form and the Health Insurance Portability and Accountability Act form. Consented participants were given a survey to complete at the time of their routinely scheduled oncology clinic visit for treatment or follow-up and allowed to return-mail the survey. Participants were compensated $10 for survey completion. Medical record data (e.g., date of diagnosis, stage of diagnosis, and treatment) were collected from the participant's medical chart.

#### 2.2.1. Measures

The survey was constructed using newly developed items grounded on information gathered during the ethnographic study and using previously existing scales such as the Cancer Worry Scale (CWS) and the Profile of Mood States (POMS) [37, 38]. Medical record data included the family history of cancer and surgery type. In addition, participants completed demographics that included age, race, ethnicity, education level, income, and residence in the federally designated Appalachian region [39]. Participants were also asked if a healthcare provider had talked to them about their risk of cancer recurrence (yes/no). The comparative risk was assessed by asking participants “Do you think your odds of getting breast cancer again are the same or different than those of other women?” consistent with previous studies [40, 41]. Respondents reported their odds on a 3-point scale from 1="lower" to 3="higher" than other women.

To understand the affective representation of cancer recurrence we used the 4-item CWS, a valid and reliable scale [38], modified to assess recurrence. For each question, respondents described their experience during the last week using a four-item Likert-type scale with response options ranging from 1 = "not at all" to 4 = "a lot". Individual scores for the four items were averaged to create a mean worry about cancer recurrence for each participant. The short form of POMS, a valid and reliable scale, was used to assess mood: depression and tension items were averaged to create a mean score for negative affect, and vigor items were averaged to create a mean score for positive effect on a scale of 1 to 5, where 1 = not at all and 5 = very much [37].

Newly constructed survey items included questions about perceived prevention and perceived control of cancer recurrence. The perceived prevention of cancer recurrence was assessed by asking participants “Do you believe you can prevent cancer recurrence (keep cancer from happening again)?” (yes/no). This question was followed by asking participants “why or why not” they responded as they did. Perceived control of progression following recurrence was assessed by asking participants “Do you believe you can control a cancer recurrence (in other words, to catch it at an early stage and/or treat it)?” (yes/no). This question was followed by asking participants “why or why not” they responded as they did.

### 2.3. Plan for Analysis

Concurrent mixed methods (qualitative and quantitative) were used to analyze the data. Using mixed methods allowed us to get an in-depth understanding of women's perception of cancer prevention and control [42]. It also allowed us to compare and contrast women's responses to the qualitative and quantitative items (i.e., triangulation). In addition, data obtained from the qualitative analysis were used in the quantitative analyses. The following analyses are listed in order of our study purpose.

#### 2.3.1. Aim 1: Quantitative Data

Descriptive statistics were used to describe our sample. To determine if perceived prevention and perceived control of cancer recurrence were seen as different, we conducted a Pearson correlation of the two variables.

#### 2.3.2. Aim 2: Qualitative Data

Using an immersion-crystallization approach [43], qualitative data helped to understand cancer survivor's lay understanding of disease by coding the survivors' response to the two open-ended questions of beliefs about prevention of cancer recurrence and beliefs about control of progression following recurrence. To begin, two research assistants independently coded 20 randomly selected participant surveys. Then, a coding framework was developed through a consensus between the two research assistants, allowing multiple codes for each participant response. For example, women report (e.g., “Based upon medical evidence, I believe it can be controlled by early detection and early treatment.”) was coded both as “catching early” and as “early treatment”, through consensus between the two research assistants. The two research assistants independently used the coding framework to code ten more randomly selected participant surveys in order to evaluate intercoder reliability using Kappa (Cohen's *κ*=0.81) [44]. Thus, these initial codes were emergent from the data. The coding framework that was developed was then classified into the five common illness attributes which included (1) identity/label (e.g., cancer in body), (2) timeline of developing cancer (e.g., acute, chronic, age of onset) (3) consequences (e.g., witness success), (4) causes (e.g., hereditary, environmental), and (5) cure/control (e.g., exercise) [45].

#### 2.3.3. Aim 3: Quantitative Data

To determine the factors associated with perceived ability to prevent and control cancer recurrence, the main outcomes were analyzed as categorical variables (binary outcome). Independent variables included age, CWS, and POMS as continuous variables. Categorical variables included race, ethnicity, education level, income, the Appalachian region, time since diagnosis (</=1 year vs. 2-5 years since diagnosis), family history of breast cancer, surgery type (lumpectomy, mastectomy), and physician communication about cancer recurrence. Two binary logistic regression models were used to predict our outcomes. Predictors that were significant at p <0.05 were retained in the final binary logistic regression models. Odds ratios of significant predictors with their 95% confidence interval were included for determining the likelihood of significant predictors. All analyses were conducted using Statistical Analysis System Software (SAS® 9.4 Institute Inc., Cary, NC, USA).

## 3. Results

### 3.1. Characteristics of the Study Sample

The mean age of participants was 58.4 (SD=10.5) years. Approximately one-quarter of respondents were from an Appalachian region [39]. Consistent with state demographics, the majority of respondents were non-Hispanic (99%) and White (96%). Thirty-six percent of participants had a high school education or less, and 55% had an annual income of more than $50,000 per year. Nearly 19% of participants reported a family history of cancer. Most participants were within one year from cancer diagnosis (55%), and around 45% are within 2-5 years from the cancer diagnosis. Sixty-five percent underwent lumpectomy, and 35% underwent mastectomy. Participants reported low mean amounts of worry (CWS: M= 1.6 (SD=0.52)). Approximately half reported higher odds of getting breast cancer versus other women; 31% reported same odds, and 21% reported lower odds. Most participants (61%) reported that a healthcare provider had talked to them about their risk of cancer recurrence.

#### 3.1.1. Aim 1: Quantitative Data

We found few women (30.0%) perceived that they could prevent cancer recurrence, and 89.1% of the women perceived they could control of progression following cancer recurrence, indicating that participants' understanding of cancer control differs from cancer prevention (Pearson r=0.09, p=0.37).  Table 1 includes sample characteristics for perceived prevention and perceived control of cancer recurrence.

#### 3.1.2. Aim 2: Qualitative Data—Cancer Prevention

In response to the open-ended question about whether or not they believed they could prevent cancer recurrence, 91.0% of participants provided an explanation (Table 1). Qualitative responses had a mean length of 14.8 words (SD=10.8). Examples of themes for perceived cancer prevention included healthy lifestyle and health behaviors, such as diet (i.e., “They should tell you sugar feeds cancer. I just read it in a book. I would have changed my diet seven years ago”) and exercise, as well as getting routine check-ups and screenings. Women also discussed their treatment features, such as having mastectomies, chemotherapy, radiation, and hormone therapy as ways to minimize recurrence. Women mentioned the hereditary predisposing factor (e.g., “I am BRCA1 and understand that getting breast cancer again is a great possibility.”). In addition, women reported spirituality and prayer as a means of prevention, healing, and coping with the threat of recurrence (e.g., “I think it's up to God and the plans for you, just give the good Lord your respect and prayer.”). Positive effect (e.g., “I definitely believe maintaining a positive outlook can help cancer recurrence.”) was also reported. However, negative effect was more typical; there was a pervasive feeling of fatalism and lack of control about preventing cancer, as they had been unsuccessful in preventing their first cancer (i.e., “We don't know when cancers happen in the first place, so it is impossible to totally prevent it from happening.”). One woman in her 1950's from Appalachia with advanced cancer sums up the sentiment that many women had about preventing cancer recurrence: “I don't think anyone knows for sure about cancer recurrence or ways to stop them! I do feel that I can do everything (almost) to put that chance at a lesser advantage. Exercise, nutrition, spirituality, feeling loved and supported, all combine to help protect.”

#### 3.1.3. Aim #2: Qualitative Data—Cancer Control

Most women (90.0%; n=88) explained their response about cancer control (Table 2). Qualitative responses had a mean length of 17.1 words (SD=15.1). Like cancer prevention, overall lifestyle, diet, and exercise were important for cancer control. The development of cancer due to factors that were beyond their control (e.g., environment, family history) was also noted. However, early detection and treatment figured more prominently in their explanations of cancer control as compared to cancer prevention (e.g., “Early detection increases your chances of winning.”). Also, monitoring for symptoms was important, a new theme for cancer control (e.g., “I feel you need to listen to your body-if it doesn't feel right…”). Another new theme emerged that was very important that seemed to empower women: witnessed the success of others (e.g., “I am living proof that it can be controlled. I have seen friends with much worse cases than mine who were completely healed.”). This was an important contrast to cancer prevention, where women felt as if they were living proof that you cannot prevent cancer. On the whole, few reported negative effect (“You can try to keep it under control, but it's not in your hands to say.”) or positive effect (“I believe a positive attitude is essential for a cancer patient… A feeling of doom and gloom is not healthy.”).

#### 3.1.4. Aim 3: Quantitative Data—Perceived Prevention of Future Cancer Recurrence

Factors associated with a greater perceived ability to prevent cancer recurrence were age, higher income, reporting identity attribute, causal attribute, consequences attribute, cure/control attribute, and lack of control (Table 3). These predictors were included in the final logistic regression model. Survivors who had reported a cause for cancer were less likely to perceive that they can prevent cancer recurrence (OR=0.11, 95% CI=0.01-0.80) (Table 4).

#### 3.1.5. Aim 3: Quantitative Data—Perceived Control of Future Cancer Recurrence

Factors associated with a greater perceived ability to control cancer recurrence included those who underwent lumpectomy, reported causal attribute, cure/control attribute, and lack of control (Table 3). These predictors were included in the final logistic regression model. Results indicated that women who had reported a cause were less likely to perceive that they can control cancer recurrence (OR=0.14, 95%CI=0.02-0.98). In addition, women that reported lack of control were less likely to perceive that they can control cancer recurrence (OR=0.12, 95%CI=0.02-0.72) (Table 4).

## 4. Discussion and Conclusions

### 4.1. Discussion

The purpose of our study was to gain a better understanding of how women with a history of breast cancer perceive their ability to prevent and control future cancer recurrence. In the case of cancer prevention, we found that when we asked women what they thought would prevent cancer; some women mentioned complementary and alternative medicine modalities of prevention such as prayer. Women also reported causes such as heredity, environment and lifestyle factors, which were similar to reported causes in other literature [30, 33]. When we asked how they might control future cancer recurrence, women reported some scientifically established interventions (e.g., healthy lifestyle) and also actions such as prayer and following medical advice. Curiously, some women responded to the quantitative item, indicating that they either did or did not believe that they could prevent or control cancer recurrence, and then went on to explain the opposite with the qualitative question. For example, they indicated they could not prevent cancer recurrence (close-ended), but then went on to explain how they could prevent cancer recurrence (open-ended).

In quantitative analysis, some of our results were similar for perceived prevention and perceived control of cancer recurrence, however there was no correlation between those outcomes in our analysis. These two concepts were distinguished in the minds of our participants, most likely a result of the wording of our questions. Most women felt they could not prevent cancer recurrence, but they did believe they could control cancer recurrence. The difference in women's perceived ability to prevention and control cancer recurrence indicates that women who have cancer may not see these two concepts as overlapping. Prevention was to keep cancer from happening again; while control meant to manage cancer once it occurs. Further, the salience and potential of control of cancer recurrence may be reinforced in the clinical setting where healthcare providers tend to focus more on clinical management and less on messages of prevention.

In this study, the causes attribute was the most consistent predictor for women's perceived ability to prevent and control cancer recurrence. In addition, "lack of control" was a significant predictor of perceived control of cancer recurrence. The finding of the association of lack of control is consistent with the wider literature showing that fatalistic beliefs are linked to delay in medical care seeking and decrease the likelihood of engaging in cancer prevention and screening behaviors [46, 47]. Sociodemographic factors (i.e., race, ethnicity, and socioeconomic status) and treatment received were not as closely related to women's perceptions about their ability to prevent and control cancer recurrence. However, as there are no existing studies to compare our findings or provide an explanation for this association, future research is warranted.

Based on our findings in the qualitative data, women were drawing on concrete, experiential knowledge of their own cancer diagnosis and treatment, as they were not able to keep cancer from occurring, but they have been able to manage the threat of cancer once it happened. However, some factors were not as closely linked to women's perceived ability to prevention and control cancer recurrence as anticipated such as cancer worry and POMs. This can be attributed to the low level of mean cancer worry and negative effect and the high level of the mean of positive effect in our sample. In the wider literature, positive effect has been linked to lower overall morbidity and mortality among breast cancer survivors. However, despite the association of positive effect with improved health outcomes, this association has not been significant with the perceived prevention and control of cancer recurrence.

Strengths and limitations of the present study deserve an explanation. First, our sample consists mostly of white, non-Hispanic, well-educated, and higher-income breast cancer survivors; thus, the study findings might differ across more heterogeneous race, ethnicity, and socioeconomic status samples. Second, our sample size was small (n=114); therefore a larger sample size is needed to provide precise estimates for the outcomes. Another limitation is the missing information about the proportion of women who consented to participate in the study; however, we estimated that approximately 90% agreed to have medical records reviewed (consented to participation), based on existing data. All measures in the study were self-reported and hence subject to recall bias. Another limitation was not using an objective measure such as the Illness Perception Questionnaire (IPQ-R) to measure the components of the patient's own representation of the illness, however, our goal was not to understand underlying lay models of cancer, our goal was to understand cancer prevention and control. In spite of these limitations, this is perhaps the first study of its kind that used qualitative and quantitative data concurrently, which elucidated women's underlying lay models of how they think they could prevent or control cancer recurrence. In addition, this is perhaps the first study that examined the difference in women's perceptions about prevention and control of cancer recurrence.

### 4.2. Conclusions

Cancer prevention and cancer control are different concepts in the minds of women with breast cancer. Women believed they could control cancer recurrence; however, few believed they could prevent it. Interventions to increase awareness about the prevention of cancer recurrence and control of progression following recurrence, focusing on evidence-based clinical and lifestyle interventions, are needed. Clinicians can play a crucial role in communicating with their patients about established clinical interventions to prevent cancer in women at high risk of cancer recurrence. Future studies are needed to examine how women's perceptions about their ability to prevent and control future cancer recurrence may affect their future engagement in health behavior.

## Figures and Tables

**Table 1:** Coded responses to open ended item of perceived prevention of cancer recurrence.

	Coded Responses for those who answered Yes		Coded Responses for those who answered No
Identity/symptoms	Not mentioned.	Identity/symptoms	Cancer in body (n=5) “I think everyone has it but depending on how your body reacts to it.”

Timeline	(i) Caught early (n=17): “if caught early, will have better outcomes.” (ii) Disease progression (n=1): “I can slow down the spread of cancer and being educated and self-awareness can help…” (iii) Early treatment (n=4): “early treatment can keep the cancer from spreading.”	Timeline	(i) First time diagnosis (n=3): “we don't know when cancers happen in the first place, so it is impossible to totally prevent it from happening.” (ii) Caught early (n= 3): “All I can do is to try catching anything early.”

Causes	(i) Environment (n= 1): “many external factors beyond our control--environment, pesticides.” (ii) Sugar (n=1): “They should tell you sugar feeds cancer. I just read it in a book. I would have changed my diet 7 years ago!” (iii) Unexpected (Unknown) (n=1): “Because based on all risk factors I shouldn't have gotten it in the first place.”	Causes	(i) Unhealthy lifestyle (n=12): “Avoid smoking, over indulging in alcohol and a reasonable amount of caffeine and diet some.” (ii) Heredity (n=6): “Both of my parents have cancer.” (iii) BRCA1 (n=1):“I am BRCA1 and understand that my getting breast cancer again is a great possibility.” (iv) Environment (n= 2): “I think there are so many external factors beyond our control--environment, pesticides, etc.” (v) Man-made carcinogen (n=1): “it's a man-made problem.” (vi) Unexpected/Unknown (n=7): “no one knows why it happens.”

Consequences	(i) Better chance of survival (n=1): “ I think that diet, exercise, a positive attitude, and scrupulous follow-up exams of your treatment team can reduce the possibility of reoccurrence, and certainly enhance the chances of survival should it reoccur…” (ii) Witness success (n=1): “… My sister had a recurrence after she stopped Tamoxifen after 5 years. She did not get chemo even though she was stage 1. I think Tamoxifen saved her, could have saved her…”	Consequences	Not mentioned.

Cure/control	(i) Healthy lifestyle (n=6): “reduce caffeine intake, better diet, increase exercise.” (ii) Avoid estrogen (n=1): “keep the estrogen out of my body.” (iii) Vitamins (n=2): “better diet, vitamins.” (iv) Follow medical advice (n=3): “by following doctor's advice.” (v) Surgery, chemotherapy, radiation therapy (n=1): “If you do the surgery, chemo, radiation, I think you prevent recurrence.” (vi) Hormonal therapy (n= 3): “I am trying with taking Tamoxifen for 5 yrs.” (vii) Regular exam (n=2): “Because of family history I plan to get regular checkups for both breast and colon cancer.” (viii) Prayer (n=2): “Prayer, diet, exercise, and vitamins, change way of living.” (ix) Knowledge (n=1): “Knowledge is power. Had I known then what I know now, I would have had an oophorectomy before age 40, rather than a hysterectomy at age 56.”	Cure/control	(i) Healthy lifestyle (n=6): “I believe you can live a better lifestyle by eating correctly, taking vitamins, and doing a lot of exercise.” (ii) Vitamins (n= 1): “I believe you can live a better lifestyle by eating correctly, taking vitamins…”

Affect	(i) Positive outlook (n= 3): “I definitely believe maintaining a positive outlook can help to prevent cancer recurrence.”	Affect	(i) Not mentioned.

Lack of Control	(i) Not mentioned.	Lack of Control	(i) Limited control (n=8): “I can delay recurrence but not prevent it.” (ii) Fatalism (n=15): “Let's just say, you probably could prevent a car accident if you drive carefully, but you can't control what someone else does with his driving or control highway conditions. You cannot control your cellular function or cancerous conditions that are present in the environment.”

**Table 2:** Coded responses to open ended item of perceived control of progression following cancer recurrence.

	Coded Responses for those who answered Yes		Coded Responses for those who answered No
Identity/ symptoms	(i) Signs/symptoms (n=4): “I feel you need to listen to your body-if it doesn't feel right.”	Identity/ symptoms	(i) Cancer in body (n=2): “Because I can't see into my body to see what is happening there; just like my heart I try to watch things but can't control all of them it is inherited I guess.”

Timeline	(i) Caught early (n=5): “if caught early, will have better outcomes.” (ii) Early diagnosis (n=3): “We all know the key to cancer survival and management is early diagnosis…” (iii) Early treatment (n=17): “the earlier you can treat cancer the better your chances of survival.” (iv) First time diagnosis: (n=1) “I'm hoping by getting regular exams it would be found soon to stop it like last time.”	Timeline	(i) Caught early (n=2): “For most cancers, the earlier they are detected the better a person's chances of survival.”

Causes	(i) Heredity (n=2): “it is inherited.”	Causes	(i) Heredity (n=1): “heredity” (ii) BRCA1 (n=1): “I am BRCA1. Controlling breast cancer is something that the doctors and I are trying…”

Consequences	(i) Witness failure (n= 1): “I've been around too many of my friends who have been very diligent and followed doctor orders, nutrition, etc. and still have died due to cancer recurrence.” (ii) Witness success (n=4): “I am living proof that it can be controlled. I have seen friends with much worse cases than mine who were completely healed. I believe a positive attitude is essential for a cancer patient- as far as controlling cancer--attitude has a lot to do with it b/c if you only thing negative-your body responds to that. A feeling of doom and gloom is not healthy for anyone.” (iii) Improve survival (n=2): “I do believe early detection is beneficial.… Catching cancer sooner before it spread to my nodes would have given me better odds.”	Consequences	(i) Witness failure (n=1): “I've seen friends who did all the right things get a recurrence somewhere else and by the time it showed up (experienced symptoms) it was too late.”

Cure/control	(i) Healthy lifestyle (n=7): “with diet and exercise it can be controlled.” (ii) Regular exam (n=3): “But, I'm hoping by getting regular exams it would be found soon to stop it like last time.” (iii) Curable (n= 14): “Yes, if in breast/breast area it is highly treatable.” (iv) Follow medical advice (n= 2): “by following doctor's advice.”	Cure/control	(i) Healthy lifestyle (n= 1): “Eating healthier, exercise.” (ii) Curable (n=1): “For most cancers, the earlier they are detected the better a person's chances of survival.” (iii) Prayer (n= 3): “eating healthier, exercise, prayer”

Affect	(i) Positive outlook (n= 7): “I believe a positive attitude is essential for a cancer patient.”	Affect	(i) Not mentioned.

Lack of Control	(i) Limited control (n=1): “You can try to keep it under control, but it's not in your hands to say.”	Lack of Control	(i) Fatalism (n=2): “Control is an illusion. (You know how to make God laugh? Tell him your plans) best to do the best you can and lie down in the boat. The river will, take you where you are supposed to go, and you will get what you need when you get there”

**Table 3:** Sample characteristics for perceived prevention of future cancer recurrence and perceived control of progression following future cancer recurrence.

Variables	Perceived Prevention^†^		Perceived Control^†^	
	Yes Number (%)	sig	Yes Number (%)	sig
Total	30 (30.0%)		90 (89.1%)	
Age in years (mean (SD))	56.4 (8.0)	*∗*	58.9 (10.5)	
Cancer Worry Scale (mean (SD))	1.58 (0.52)		1.63 (0.5)	
Negative affect (mean (SD))	1.98 (0.68)		1.8 (0.7)	
Positive Affect (mean (SD))	3.2 (0.84)		3.3 (0.9)	
Race				
White	29 (96.7%)		87(96.7%)	
Other	1 (3.3%)		3 (3.3%)	
Education Level				
High school or less	7 (23.3%)		33 (91.7%)	
Some college or 2-year college	7 (23.3%)		22 (91.7%)	
4-year degree	7 (30.1%)		17 (85.0%)	
Graduate degree	9 (42.9%)		18 (85.7%)	
Income		*∗*		
less than 15,000	2 (7.1%)		8 (9.3%)	
15,000 to 49,999	4 (14.3%)		30 (34.9%)	
More than 50,000	22 (78.6%)		48 (55.8%)	
Appalachian				
Yes	3 (10.3%)		19 (21.8%)	
No	26 (89.7%)		68 (77.0%)	
Time since diagnosis				
Within 1 year	14 (46.7%)		50 (55.6%)	
2-5 years	16 (53.3%)		40 (44.4%)	
Family History of Cancer				
Yes	4 (13.3%)		16 (17.8%)	
No	26 (86.7%)		74 (82.2%)	
Lumpectomy				*∗*
Yes	18 (64.3%)		59 (68.6%)	
No	10 (35.7%)		27 (31.4%)	
Mastectomy				
Yes	12 (42.9%)		32 (37.2%)	
No	16 (57.1%)		54 (62.3%)	
Physician communication				
Yes	21 (70.0%)		57 (64.8%)	
No	9 (30.0%)		31 (35.2%)	
Attributes of Cancer				
Identity/Symptoms		*∗*		
Yes	0 (0.0%)		1 (1.1%)	
No	28 (100.0%)		89 (98.9%)	
Timeline				
Yes	3 (7.4%)		35 (45.5%)	
No	25 (92.6%)		42 (54.5%)	
Cause		*∗*		*∗*
Yes	5 (16.7%)		4 (5.2%)	
No	25 (83.3%)		73 (94.8%)	
Consequence		*∗*		
Yes	2 (7.3%)		4 (5.2%)	
No	26 (92.7%)		73 (94.8%)	
Cure/Control		*∗*		
Yes	25 (96.2%)		61 (79.2%)	
No	3 (8.2%)		16 (20.8%)	
Lack of Control				*∗*
Yes	1 (8.2%)		5 (6.7%)	
No	26 (96.2%)		70 (93.3%)	

^†^Reported numbers and percentage are relative to the column attribute. *∗* P < 0. 05.

**Table 4:** Odds ratios and 95% confidence intervals from multinomial logistic regression on perceived cancer prevention and perceived control of cancer progression.

Variables	Perceived Prevention of Cancer			Perceived Control of Cancer Progression		
	OR	95% CI	Sig	OR	95% CI	Sig
Income						
≥ 50,000 (Ref.)						
<15,000	-2.31	0.01-2.25		-4.31	0.07-6.25	
15,000 to 49,999	-1.16	0.04-1.25		-1.16	0.03-2.06	
Lumpectomy						
No (Ref.)	3.31	0.68-5.8		4.8	0.57-6.45	
Yes	1.16	0.04-1.25		2.65	0.08-7.06	
Causal Attribute						
No (Ref.)						
Yes	0.11	0.01-0.80	*∗*	0.14	0.02-0.98	*∗*
Lack of Control						
No (Ref.)						
Yes	4.08	0.54-30.85		0.12	0.02-0.72	*∗*

Note: based on 114 women with a history of breast cancer and 18 years of age or older. Asterisks represent significant group differences compared to the reference group based on the binary logistic Regression.

OR: odds ratio, CI: confidence interval, Sig: significance, and Ref: reference. *∗* P < .05.

## Data Availability

Deidentified data are available upon Institutional Review Board approval.
